# Sleeping Sound Autism Spectrum Disorder (ASD): Cost-Effectiveness of a Brief Behavioural Sleep Intervention in Primary School-Aged Autistic Children

**DOI:** 10.1007/s10803-024-06422-2

**Published:** 2024-06-04

**Authors:** Lidia Engel, Oxana Chiotelis, Nicole Papadopoulos, Harriet Hiscock, Patricia Howlin, Jane McGillivray, Susannah T. Bellows, Nicole Rinehart, Cathrine Mihalopoulos

**Affiliations:** 1 Monash University Health Economics Group, School of Public Health and Preventive Medicine, Monash University, Melbourne, VIC Australia; 2 Deakin Health Economics, School of Health and Social Development, Deakin University, Geelong, VIC Australia; 3 Krongold Clinic, Faculty of Education, Monash University, Notting Hill, VIC Australia; 4 School of Educational Psychology & Counselling, Monash University, Melbourne, VIC Australia; 5 Murdoch Children’s Research Institute, The Royal Children’s Hospital, Parkville, VIC Australia; 6 Department of Paediatrics, University of Melbourne, Parkville, VIC Australia; 7 Institute of Psychiatry, Psychology and Neuroscience, King’s College London, London, UK; 8 School of Psychology, Faculty of Health, Deakin University, Geelong, VIC Australia

**Keywords:** Cost-effectiveness, Autism, Sleep, Children, Quality-adjusted life years

## Abstract

Disordered sleep is common in autistic children. This study aimed to evaluate the cost-effectiveness of a brief behavioural sleep intervention, the ‘*Sleeping Sound* intervention’, in primary school-aged autistic children in Australia. A cost-effectiveness analysis was undertaken alongside a randomised controlled trial over a 6-month follow-up period from both a societal and healthcare sector perspective. Resources used by participants were collected from a resource-use questionnaire and administrative data; intervention costs were determined from study records. Mean costs and quality-adjusted life-years (QALYs) were compared between the intervention and treatment as usual (TAU) groups. Uncertainty analysis using bootstrapping and sensitivity analyses were conducted. The sample included 245 children, with 123 participants randomised to the intervention group and 122 to TAU. The mean total costs were higher for the *Sleeping Sound* intervention with a mean difference of A$745 (95% CI 248; 1242; p = 0.003) from a healthcare sector perspective and A$1310 (95% CI 584; 2035, p < 0.001) from a societal perspective. However, the intervention also resulted in greater QALYs compared with TAU, with a mean difference of 0.038 (95% CI 0.004; 0.072; *p* = 0.028). The incremental cost-effectiveness ratio was A$24,419/QALY (95% CI 23,135; 25,703) from a healthcare sector perspective and A$41,922/QALY (95% CI 39,915; 43,928) from a societal perspective; with a probability of being cost-effective of 93.8% and 74.7%, respectively. Findings remained robust in the sensitivity analyses. The *Sleeping Sound* intervention offers a cost-effective approach in improving sleep in primary school-aged autistic children.

*Trial registration* The trial was registered with the International Trial Registry (ISRCTN14077107).

**Supplementary Information:**

The online version contains supplementary material available at 10.1007/s10803-024-06422-2.

## Introduction

Autism spectrum disorder (ASD)^1^ is a neurodevelopmental disorder, characterised by difficulties in social communication and social interaction and the presence of restricted and repetitive behaviours (American Psychiatric Association, 2013). The global prevalence of autism is estimated to be at least 1% (Bougeard et al., 2021; Zeidan et al., 2022). In Australia, the number of individuals with autism has increased significantly in recent years, from estimated 64,400 people in 2009 to 164,000 in 2015, which corresponds to 0.7% of the Australian population (Australian Institute of Health & Welfare, 2017). Often identified in children and young people, 83% of individuals with autism are under the age of 25 years (Australian Institute of Health & Welfare, 2017). The economic burden of childhood ASD is substantial. In Australia, the economic costs of ASD range between A$8.1 and A$11.2 billion annually in 2011 (Synergies Economic Consulting, 2011), with the median family cost estimated to be A$34,900 per annum, with almost 90% of the sum (A$29,000) due to loss of income from employment reported by parents (Horlin et al., 2014).

Sleep problems are among the most typical difficulties in autistic children, affecting about 40–80% of those during their childhood (Cohen et al., 2014; Malow et al., 2014). Sleep problems are usually behavioural and common problems include sleep-onset delay, shorter sleep duration, frequent prolonged night wakings, bedtime resistance, sleep anxiety, daytime sleepiness, early waking, co-sleeping, low sleep efficiency and parasomnias (Richdale & Schreck, 2009). Sleep problems in autistic children are associated with poorer child functioning, including increased autism symptom severity (Tudor et al., 2012), increased behavioural problems (Mazurek & Sohl, 2016) and poorer health-related quality of life (Delahaye et al., 2014). Sleep problems in autistic children have also been linked to poor parental mental and physical health as well as parenting stress (Hodge et al., 2014; Lovell et al., 2021; Martin et al., 2021; Tilford et al., 2015).

Sleep problems in autism are commonly managed with behavioural and pharmacological interventions (Beresford et al., 2018). However, the aetiology of sleeping problems in autistic children is likely multifactorial and may include biopsychosocial factors (Richdale & Schreck, 2009). While behavioural sleep interventions appear promising, the empirical evidence for their efficacy for autistic children is inconclusive (Beresford et al., 2018; Vriend et al., 2011). Previous studies that examined the efficacy of behavioural sleep interventions have suffered from inadequate sample size, design limitations and lack of clarity in defining whether behavioural sleep interventions lead to improvements in other domains of functioning, such as child behavioural and emotional problems, social communication, parenting stress and parent mental health (Pattison et al., 2020).

A recent randomised controlled trial (RCT) conducted in Australia that included 245 primary school-aged autistic children examined the efficacy of a brief behavioural sleep intervention, hereafter ‘*Sleeping Sound* intervention’ (Papadopoulos et al., 2022). The intervention consisted of two 50-min face-to-face sessions plus a follow-up phone call by a trained clinician. The study found that parents/caregivers in the intervention group (n = 123) reported a reduction in child sleep problems at 3 months post-randomisation (effect size: − 0.7) compared with the treatment as usual group (n = 122). The intervention was reported to be acceptable and feasible by families/caregivers (Pattison et al., 2022a). Small effects were also observed for a number of secondary child and parent outcome measures, albeit not significant after adjusting for multiple comparisons. Although the *Sleeping Sound* intervention is efficacious, with longer term benefits also reported at 12-months follow up (Pattison et al., 2022b), and has the potential to be easily embedded into the Australian healthcare system, it remains unknown whether the intervention also represents ‘good value for money’.

Economic evaluation is a tool used to provide decision-makers with evidence regarding which interventions or services provide good value for money or are cost-effective. Economic evaluation is defined as a comparative analysis of alternative courses of action in terms of both their costs and consequences (Drummond et al., 2015). To date, there is a lack of evidence on the cost-effectiveness of treatments for autistic children and adolescents (Pye et al., 2023; Sampaio et al., 2021). This may be driven by unaddressed challenges in conducting economic evaluations of autism interventions related to diversity of autism presentation, and difficulty with measuring costs and outcomes, including measurement of family effects (Tsiplova & Ungar, 2023). Given the paucity of cost-effectiveness evidence in general, the Lancet Commission on the future of care and clinical research in autism has recently highlighted the need for more health economic analysis (Lord et al., 2022).

Currently, the cost-effectiveness of behavioural sleep interventions remains unknown. This paper describes the economic evaluation of the behavioural sleep program, *Sleeping Sound* intervention, using resource use and outcome data collected from the *Sleeping Sound* RCT participants. The program was delivered by study clinicians to treat sleep problems in autistic children aged 5–13 years. The research question was whether the *Sleeping Sound interventio*n is cost-effective compared to treatment as usual (TAU) over a 6-months follow-up period from both societal and healthcare sector perspectives in terms of improvement in quality-adjusted life-years (QALYs).

## Methods

The study protocol has been published elsewhere (Papadopoulos et al., 2019) and the trial was registered with the International Trial Registry (ISRCTN14077107). Ethics was granted from the Human Research Ethics Committees from the Royal Children’s Hospital, Melbourne (36154), Deakin University (2017-130), Victorian Department of Education and Early Childhood Development (2016_003134) and the Catholic Education Office Melbourne (0501). In reporting the design and findings of our economic evaluation, we adhere to the 2022 Consolidated Health Economic Evaluation Reporting Standards (Husereau et al., 2022) presented in Supplementary Table 1.

### Trial Design and Participants

This study was conducted in Melbourne, Australia, and targeted families of autistic children, aged 5–13 years, who were attending primary school at the time of recruitment (Papadopoulos et al., 2022). Recruitment of children was conducted between May 2017 and March 2019 according to referrals from Victorian paediatric clinics and by advertising in clinical, research, and community networks. Upon completion of consent forms and baseline surveys, participants were randomised by an independent research assistant into either the intervention or TAU group. To be eligible, all children had: (i) written evidence of a clinically confirmed, DSM IV or DSM-5 multidisciplinary diagnosis of ASD or confirmation by the treating paediatrician; (ii) clinical cut-off score ≥ 11 for ASD symptom severity on the Social Communication Questionnaire–Lifetime form (Rutter et al., 2003); (iii) a parent/caregiver-reported moderate-to severe behavioural sleep problem persisting for ≥ 4 weeks; and (iv) at least one parent/caregiver-reported child sleep problem for chronic insomnia and/or delayed sleep–wake phase as defined by the International Classification of Sleep Disorders—Third-Edition diagnostic criteria (American Academy of Sleep Medicine, 2014). Children with intellectual disability, co-occurring medical condition known to impair sleep, genetic conditions related to intellectual impairment, or suspected obstructive sleep apnoea were excluded.

### Interventions

The *Sleeping Sound* intervention consisted of two consecutive 50-min face-to-face sessions provided to children and their parents/caregivers and a follow-up phone call after the second face-to-face session, all at 2-week intervals, delivered by a clinician (e.g., paediatrician or psychologist) (Papadopoulos et al., 2022). The intervention sessions focused on: (i) assessing the type and likely cause of the child’s specific sleep difficulties; (ii) provision of psychoeducation about normal sleep patterns and identifying the child’s specific sleep problem(s), as well as developing healthy sleep practices; (iii) tailored strategies to develop an individualised sleep management plan, and (iv) visual tools and written information sheets for parents to facilitate delivery of the intervention (Papadopoulos et al., 2022). Families allocated to the TAU group received typically available web-based ‘tip sheets’ related to the topic of sleep management for autistic children.

### Outcomes

Child outcomes were measured in terms of QALYs that combine length of life and quality of life in a single index. For children aged 5–7 years, proxy assessment via parent/guardian is recommended for quality of life data collection (Mpundu-Kaambwa et al., 2022). In this study, the Child Health Utility 9D (CHU9D) questionnaire was completed by parents regardless of the child’s age as proxy-report at baseline, 3-month and 6-month follow-up and QALYs were generated applying the area-under-the-curve approach (Drummond et al., 2015). The CHU9D is a paediatric generic preference-based quality of life measure developed for use in economic evaluation for children aged 7–11 years but has also been used in adolescents aged 12–18 years (Rowen et al., 2021). It contains nine dimensions (worried, sad, pain, tired, annoyed, schoolwork/homework, sleep, daily routine, and activities), each with five severity levels. The scoring of the CHU9D for this study was based on the Australian tariff (Ratcliffe et al., 2016). The resulting health utility scores are anchored on a 0 (dead) to 1 (full health) scale, with negative scores denoting states that are considered worse than being dead. The CHU9D has been widely used in different countries across different conditions with several papers providing support for its validity (Rowen et al., 2021).

Previous literature suggests that healthcare interventions that improve the health of autistic children, also have the potential to affect the health of caregivers/parents (Brown et al., 2019; Tilford et al., 2015). This is often referred to as ‘spillover effects’ (Wittenberg et al., 2019). A study evaluating a sleep treatment for children with autism found that the spillover QALYs gained by caregiver were of similar magnitude as the gains to the child (Tilford et al., 2015). In our study, we measured spillover QALYs using the Assessment of Quality of Life 4-dimension (AQoL-4D), which has 12 items that describe four dimensions (independent living, social relationships physical senses, and psychological wellbeing); each item is rated on a 4-point response scale (Hawthorne et al., 2001). Utility scores were generated using the Australian value set of the AQoL-4D and spillover QALYs were generated using the area under the curve approach. However, given existing methodological shortcomings in incorporating spillover effects in economic evaluations (Dixon & Round, 2019; McCaffrey et al., 2020; Wittenberg et al., 2019), spillover QALYs were only considered in a sensitivity analysis (see below).

### Resource Use and Costs

The economic evaluation was conducted from a societal and a healthcare sector perspective, as recommended by The Second Panel on Cost-Effectiveness in Health and Medicine (Sanders et al., 2016). Healthcare sector costs included cost associated with the child’s healthcare resources use, such as visits to health professionals, as well as pharmaceuticals, hospital services (emergency department visit, hospital admissions, and ambulance use), and out of pocket costs related to health services. Societal cost included travel cost and parental lost productivity (i.e., absenteeism) to care for their child. Supplementary Table 2 contains an impact inventory with detailed cost components included by the respective perspective. A Resource Use Questionnaire (RUQ) was completed by parents/caregivers at baseline, 3-months and 6-months follow-up, which referred to services used for child’s physical health, mental health and educational support. In addition, parents were asked for their consent to access their child’s Medicare data (Medicare Benefits Scheme, MBS) and Pharmaceutical Benefits Scheme (PBS) data for a period of 3 months prior to baseline up until 6-months post randomisation. The MBS and PBS information were supplied by Services Australia and were only used in a sensitivity analysis. Resource use data as well as MBS and PBS data covering the period prior to randomization were used to control for baseline differences between treatment groups (van Asselt et al., 2009).

All health professional services were costed using a weighted average national cost paid by the government for the corresponding health professional, derived from the MBS item reports (Australian Government, 2016a). Medication cost were estimated by using PBS item prices, which also included out-of-pocket cost (Australian Government, 2016b). Online Australian retail pharmacy sites were accessed to determine patient costs for other medications and supplements not covered by the PBS. Hospital stays and emergency department visits were costed using the National Hospital Cost Data Collection cost report (Round 21) (Independent Hospital Pricing Authority, 2019). Travel costs for parents to attend healthcare services were calculated from the distance travelled in kilometres reported in the RUQ and multiplied by the unit cost, depending on the type of transport used (car, public transport, ambulance). Time taken off from paid work was valued by using an average hourly wage rate calculated from the average weekly earnings reported by the Australian Bureau of Statistics, plus 25% overhead costs (Australian Bureau of Statistics, 2016). Time off from unpaid activities was valued at 25% of the average wage rate to represent the value of participants’ lost leisure time (Jacobs & Fassbender, 1998). Supplementary Tables 3 and 4 list the unit costs used. All costs are presented in Australian dollars (A$) for the 2016–2017 financial reference year, reflecting the time point when the study commenced. Since the costs and outcomes were collected over a 6-month period, discounting was not applied.

### Intervention Costs

The cost of the *Sleeping Sound* intervention was calculated using a micro-costing approach from both a healthcare sector perspective and a societal perspective. Resources used to develop and deliver the intervention were obtained from study records. Consequently, intervention cost calculation employed two scenarios; one based on actual intervention delivery time and one assuming full adherence and intended time for intervention delivery. Personnel costs included two three-hour training sessions delivered by a clinical psychologist to three general psychologists. Rates for a clinical psychologist (A$200 per hour) and general psychologist (A$185 per hour) were obtained from the Australian Psychological Society (Australian Psychological Society, 2021). Intervention delivery comprised two 50-min face-to-face sessions (plus 10-min preparation time), in addition to clinician 45-min travel time and travel cost (30 km round-trip on average), costed at A$0.66 per km (Australian Government, 2022). The actual time to deliver the first session was estimated from study records to be 52 min and 33 min for the second session. The cost for the 30-min follow-up phone call (17-min actual time) incorporated also scheduling time and reminder text messages sent to study participants. Intervention cost further comprised printing cost and preparation of manuals/folders for clinicians and participants. The development of the *Sleeping Sound* intervention content, which included personnel cost and cost for the graphic designer, was excluded from the base case analysis, as these are considered sunk cost (i.e., a cost that has already been incurred and cannot be recovered). In the sensitivity analysis, an average cost of A$0.76 was added to each participant in the intervention group, which reflects the true cost per participant once the development costs have been distributed to all autistic children aged 5–13 years in Australia. Costs for parents were only considered when adopting a societal perspective that included parent time to attend all sessions and travel time (costed at the national average wage rate), travel cost, parking and parent time to complete a 5-min homework task. Further details about the intervention cost can be obtained from the Supplementary Table 5. For the primary analysis, intervention costs were based on the actual delivery time (healthcare sector perspective: A$1079; societal perspective: A$1333—per participant); sensitivity analyses explored the cost-effectiveness of *Sleeping Sound* intervention using estimates for intended intervention delivery time (healthcare sector perspective: A$1282; societal perspective: A$1536 per participant). These intervention costs translate to US$925 and US$1143 (US$1099 and US$1317 in the sensitivity analysis) in 2024.

### Statistical Analysis

Statistical analyses were performed in Stata version 17. The primary analysis was undertaken according to intention-to-treat (ITT). A descriptive analysis of missing data that included an exploration of missing values patterns was performed. Supplementary Table 6 shows the proportion of missing values in costs and outcomes by treatment allocation. Missing values for the TAU group were slightly higher compared to the intervention group at 3-months (21% vs 17%) and 6-months follow-up (28% vs 27%) for both costs and utilities. As for MBS and PBS data, 28% and 36% from the intervention group and 36% and 41% from the TAU group did not consent to the use of their MBS and PBS data, respectively. Mean imputation was applied to impute missing baseline utility scores. Multiple imputation was performed to handle missing data for all cost variables by chained equations (MICE), whereby data were imputed 50 times. The imputation model included utility values for the child and parent/caregiver at all time points, child and parent characteristics (age and gender), broad RUQ cost categories (health professionals; pharmaceuticals; out-of-pocket cost; hospital services; productivity cost, and travel cost) MBS, PBS and intervention cost.

Outcomes and costs were compared between the TAU group and intervention group using Ordinary Least Squares regression (OLS) and generalized linear models (GLM) from both the healthcare sector and societal perspectives. For mean differences in total costs, a gamma distribution and a log link were applied, whereas for the differences in QALYs, a Gaussian distribution and identity link was used. Analyses were also conducted for all cost components and MBS and PBS data. Both unadjusted and adjusted models that included baseline utilities, baseline costs, children and parents gender, and children’s age were performed.

Incremental cost-effectiveness ratios (ICERs) were calculated as the difference in cost divided by the difference in the outcome between the two study arms. ICER was presented from a societal and a healthcare sector perspective. To reflect sampling uncertainty, a nonparametric bootstrap procedure with 1000 iterations was employed to estimate the confidence intervals around the ICER. A cost-effectiveness plane, which is a plot of the 1000 bootstrapped incremental costs and outcomes across four quadrants was presented to graphically demonstrate the bootstrapped ICERs and the CIs. In addition, a cost-effectiveness acceptability curve was constructed by calculating the probability of the intervention being cost-effective at different values of willingness-to-pay. While Australia does not have an explicit ICER threshold, an implicit threshold of A$50,000/QALY was assumed below which an intervention is considered cost-effective (Wang et al., 2018).

### Sensitivity Analysis

Sensitivity analyses were performed to examine the robustness of the results. First, base case results were compared to the complete data results. Second analysis accounted for spillover QALYs, where the ICER was recalculated based on total QALYs that summed up child QALYs and parental QALYs. The third sensitivity analysis replaced some RUQ data (i.e., health professional services and pharmaceuticals) with MBS and PBS data, and the final sensitivity analysis used intervention cost based on intended session delivery times (healthcare sector perspective: A$1282; societal perspective: A$1536 per participant).

## Results

The sample included 245 participants, with 123 participants randomised to the intervention group and 122 to the TAU group. Baseline characteristics of children and their parents/caregivers are displayed in Table 1, showing that the TAU and intervention group were well matched in terms of age, gender, autism severity and baseline utility scores.

**Table 1:** Baseline characteristics of the study population, n (%)

	Intervention group (n = 123)	TAU (n = 122)
Children		
Age in years, mean (SD)	8.45 (2.1)	9.18 (2.1)
Gender		
Male	80 (65%)	81 (66%)
Female	43 (35%)	41 (34%)
ASD symptom severity, mean (SD)	14.11 (5.82)	14.65 (5.69)
Parent-reported comorbidities		
Anxiety disorder	53 (43%)	63 (52%)
Attention deficit/hyperactivity disorder	44 (36%)	52 (43%)
Oppositional defiant disorder	10 (8%)	14 (11%)
Depressive disorder	2 (2%)	2 (2%)
CHU9D, mean (SD)	0.526 (0.017)	0.520 (0.017)
Primary caregiver		
Age in years, mean (SD)	41.02 (4.80)	41.87 (5.52)
Female	119 (97%)	115 (94%)
Education		
Did not complete high school	11 (9%)	15 (12%)
Completed high school only	29 (24%)	30 (25%)
Completed tertiary study	83 (67%)	77 (63%)
Single parent household	23 (19%)	34 (28%)
AQoL-4D, mean (SD)	0.692 (0.019)	0.675 (0.021)

Table 2 provides the mean cost and outcomes by treatment allocation. Compared with the TAU group, the *Sleeping Sound* intervention resulted in lower cost for health professional visits, pharmaceuticals, and out-of-pocket costs but higher costs for hospital services, work absenteeism and travel cost. However, statistical significance was only reached for pharmaceutical cost (*p* = 0.025). The mean total costs from both healthcare sector and societal perspectives were estimated to be higher for the *Sleeping Sound* intervention arm, when the costs of the intervention were included, with a mean difference of A$745.12 (t = 2.95; 95% CI 247.8; 1242.47; p = 0.003) from a healthcare sector perspective and A$1309.5 (t = 3.56; 95% CI 584.15; 2034.8, p < 0.001) from a societal perspective, respectively. The findings were statistically significant and remained significant after adjusting for baseline cost. In terms of outcomes, mean QALYs were higher for the intervention arm (0.2997; SE: 0.008) when compared with the TAU group (0.262; SE: 0.015) with a mean difference of 0.038 (t = 2.21; 95% CI 0.004; 0.072; *p* = 0.028). The mean difference remained significant even after adjusting for baseline utilities (*p* = 0.021). No significant difference (*p* = 0.428) was found for parent/caregiver QALYs between the intervention (0.351; SE: 0.010) and the TAU group (0.337; SE: 0.013).

**Table 2:** Mean cost and outcomes by treatment allocation (after multiple imputation)

Cost items	Intervention (n = 123)		TAU (n = 122)		MD	SE	95% CI	p-value
	Mean	SE	Mean	SE				
Intervention cost per participant (HP)	1079	–	0	–	–	–	–	–
Intervention cost per participant (SP)	1333	–	0	–	–	–	–	–
Health professionals	812.48	95.69	937.41	98.92	− 124.93	138.68	− 398.51; 148.66	0.369
Pharmaceuticals	106.55	20.27	184.12	27.85	− 77.57	34.32	− 145.2; − 9.94	0.025
Hospital services	189.38	78.51	151.17	98.87	38.21	126.29	− 210.58; 286.99	0.763
Out-of-pocket	928.07	126.52	1127.04	142.78	− 198.97	190.65	− 574.83; 176.88	0.298
Absenteeism	1133.9	178.4	846.64	135.72	287.26	226.3	− 158.89; 733.4	0.206
Travel	4.68	1.36	2.69	0.85	1.99	1.6	− 1.16; 5.14	0.215
*Total healthcare sector cost*	3133.53	164.13	2388.41	197.27	745.12	252.36	247.8; 1242.47	0.003
*Total societal cost*	4545.64	267.32	3236.16	254.37	1309.5	367.87	584.15; 2034.8	< 0.001
CHU9D baseline	0.526	0.017	0.520	0.017	0.006	0.024	− .041; 0.05	0.806
CHU9D 3 months	0.627	0.021	0.506	0.054	0.121	0.057	0.008; 0.23	0.036
CHU9D 6 months	0.618	0.025	0.562	0.026	0.056	0.036	− .014; 0.13	0.117
*QALYs-child*	0.2997	0.008	0.262	0.015	0.038	0.017	0.004; 0.072	0.028
AQoL-4D baseline	0.692	0.019	0.674	0.021	0.018	0.029	− 0.04; 0.07	0.532
AQoL-4D 3 months	0.705	0.025	0.66	0.045	0.046	0.052	− 0.06; 0.15	0.386
AQoL-4D 6 months	0.704	0.022	0.705	0.026	− 0.001	0.034	− 0.068; 0.07	0.967
*QALYs-parent*	0.351	0.010	0.337	0.013	0.013	0.017	− 0.02; 0.047	0.428

*HP* healthcare sector perspective, *SP* societal perspective, *MD* mean difference, *SE* standard error, *95%CI* 95% confidence interval

**Fig. 1:**
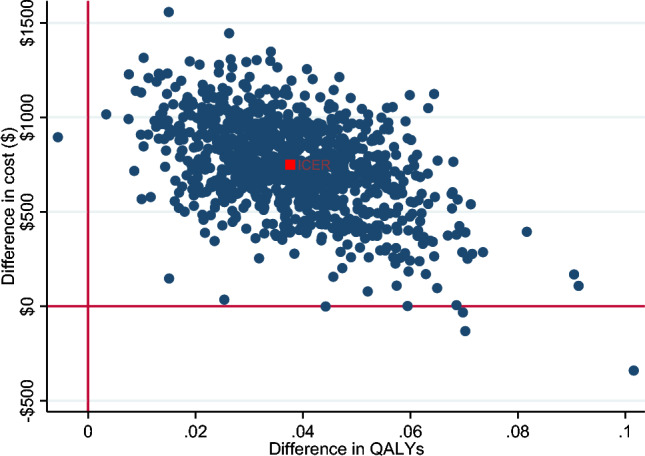
Cost-effectiveness plane (healthcare perspective). *Note* In the north-east quadrant, the intervention is cost-effective if the ICER falls under the specified value-for-money criterion since the intervention is more effective and more costly than the comparator. In the south-east quadrant, the intervention is less costly and more effective than the comparator (i.e. dominant), therefore the intervention is likely to be excellent value-for-money. In the south-west quadrant, the intervention is less costly and less effective, therefore the decision to adopt the intervention may be based on decision-makers willingness to accept some health loss relative to cost-saving. Finally, in the north-west quadrant, the results show the intervention are associated with greater costs but less health gain, therefore, not a good option to adopt (referred to as dominated)

**Fig. 2:**
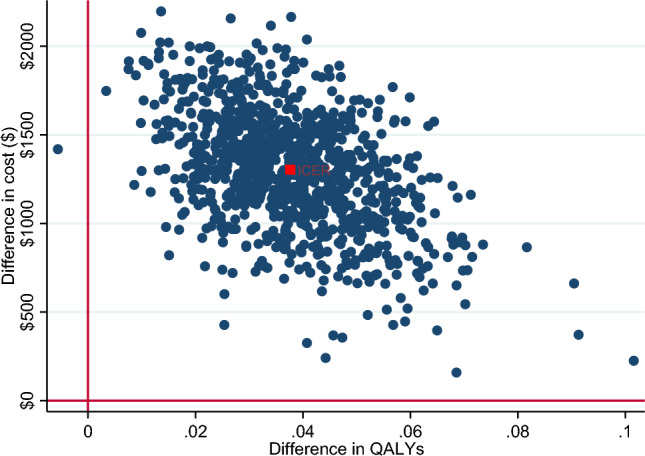
Cost-effectiveness plane (societal perspective). *Note* In the north-east quadrant, the intervention is cost-effective if the ICER falls under the specified value-for-money criterion since the intervention is more effective and more costly than the comparator. In the south-east quadrant, the intervention is less costly and more effective than the comparator (i.e. dominant), therefore the intervention is likely to be excellent value-for-money. In the south-west quadrant, the intervention is less costly and less effective, therefore the decision to adopt the intervention may be based on decision-makers willingness to accept some health loss relative to cost-saving. Finally, in the north-west quadrant, the results show the intervention are associated with greater costs but less health gain, therefore, not a good option to adopt (referred to as dominated)

**Fig. 3:**
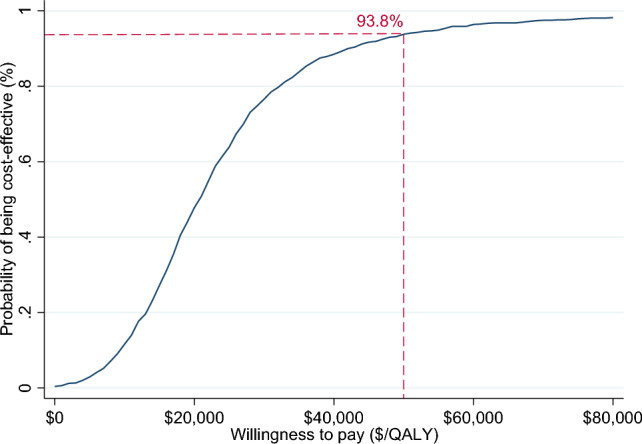
Cost-effectiveness acceptability curve (healthcare perspective)

**Fig. 4:**
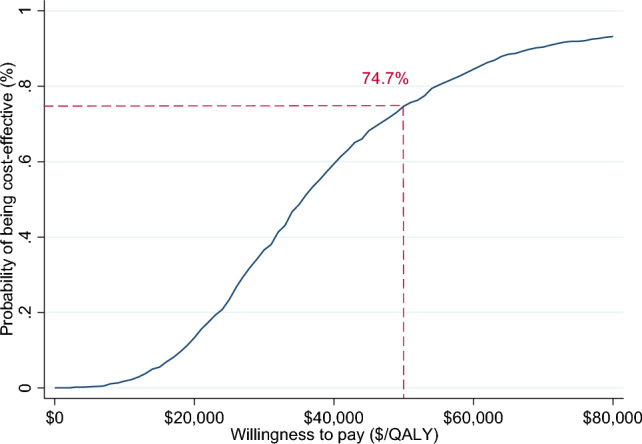
Cost-effectiveness acceptability curve (societal perspective)

Bootstrapped incremental costs, outcomes and ICERs are provided in Table 3, indicating that *Sleeping Sound* intervention resulted in greater cost but also greater QALY gains, with 99% of the bootstrapped iterations sitting in the north-east quadrant (see Figs. 1 and 2). The ICER was A$24,297.03/QALY (95% CI 23,075.57; 25,518.49) from a healthcare sector perspective and A$41,921.63/QALY (95% CI 39,915.07; 43,928.2) from a societal perspective. Using the A$50,000 per QALY threshold that is commonly applied in Australia (Wang et al., 2018), the *Sleeping Sound* intervention had a 93.8% and 74.7% probability of falling below this threshold from the healthcare sector and societal perspectives, respectively. This is illustrated in the cost-effectiveness acceptability curves in Figs. 3 and 4.

**Table 3:** Bootstrapped incremental cost and outcomes, ICER and distribution of bootstrapped iterations on cost-effectiveness plane

Perspective	Incremental cost (95% CI)^a^	Incremental QALYs (95% CI)^b^	ICER (95% CI)	NE (%)	NW (%)	SW	SE	Probability cost-effective ($50,000) (%)
Healthcare	$749.83 (735.12; 764.54)	0.038 (0.037; 0.038)	$24,297.03 (23,075.57; 25,518.49)	99.5	0.1	0	0.4%	93.8
Societal	$1303.4 (1283.63; 1323.53)	0.038 (0.037; 0.038)	$41,921.63 (39,915.07; 43,928.2)	99.9	0.1	0	0	74.7

*ICER* incremental cost-effectiveness ratio, *NE* north-east quadrant, *NW* north-west quadrant, *SW* south-west quadrant, *SE* south-east quadrant

^a^Costs adjusted for baseline cost

^b^QALYS adjusted for baseline utilities

Findings from the sensitivity analyses are shown in Table 4, highlighting that overall the intervention remained cost-effective under different circumstances. The exemption was when using complete data instead of imputed data, where the ICER slightly exceeded the A$50,000 threshold mark when adopting a societal perspective (ICER of A$59,176.67/QALY). Replacing children QALYs by total QALYs, which combined child and parent/caregiver QALYS, resulted in a significant reduction in ICER from both perspectives. Replacing the intervention cost using the intended delivery time rather than the actual intervention delivery time did not appreciably change the results. Replacing RUQ costs related to health professionals’ services use and pharmaceuticals consumption with MBS and PBS data respectively, resulted in higher ICER from both perspectives but still remained under the threshold of A$50,000.

**Table 4:** Sensitivity analysis results

	Costs (mean)		QALYs (mean)		∆Cost	∆QALY	ICER
	Intervention	TAU	Intervention	TAU			
Base case							
Healthcare (n = 245)	3133.53	2388.41	0.2997	0.262	745.12	0.038	19,608.4
Societal (n = 245)	4545.64	3226.16	0.2997	0.262	1309.5	0.038	34,460.5
Complete case							
Healthcare (n = 166)	3395.26	2320.4	0.305	0.275	1074.8	0.03	35,826.7
Societal (n = 166)	4982.1	3206.8	0.305	0.275	1775.3	0.03	59,176.7
Total QALYs							
Healthcare (n = 245)	3133.53	2388.41	0.650	0.599	745.12	0.051	14,610.2
Societal (n = 245)	4545.64	3226.16	0.650	0.599	1309.5	0.051	25,676.5
Different intervention cost							
Healthcare (n = 245)	3336.53	2388.41	0.2997	0.262	948.12	0.038	24,950.5
Societal (n = 245)	4748.64	3236.16	0.2997	0.262	1512.5	0.038	39,802.6
MBS/PBS data							
Healthcare (n = 245)	1839.43	743.86	0.2997	0.262	1095.6	0.038	28,831.6
Societal (n = 245)	3251.54	1591.6	0.2997	0.262	1659.94	0.038	43,682.6

*MBS* Medicare benefits schedule, *PBS* pharmaceuticals benefits scheme, *QALYs* quality adjusted life years, *∆Cost* incremental cost, *∆QALY* incremental QALY, *ICER* incremental cost-effectiveness ratio

## Discussion

This study reports findings from the first economic evaluation of a brief behavioural sleep intervention aimed at improving sleep in primary school-aged autistic children. Data were based on the largest published RCT of a brief behavioural intervention for autistic children. Findings show that children allocated to the *Sleeping Sound* intervention accrued greater cost but also more QALYs. Using an implicit threshold of A$50,000/QALY, the probability of the intervention being cost-effective was 93.8% and 74.7% from the healthcare sector and societal perspectives, respectively. Our findings remained robust when testing different scenarios in the sensitivity analyses. These results complement findings from the RCT, showing that the *Sleeping Sound* intervention is not only effective in improving sleep in primary school-aged autistic children but also represents good value for money.

The costs of the *Sleeping Sound* intervention (healthcare sector perspective: A$1079; societal perspective: A$1333 per participant) are similar to five occupational therapy, speech therapy or behavioural support sessions (A$214.41 per hour) in Australia (Autism Spectrum Australia (Aspect), 2023). The intervention was shown to reduce visits to health professionals, use of pharmaceuticals and out-of-pocket costs associated with healthcare services. While total costs were higher for the intervention group compared with the TAU group from both perspectives, the intervention was slightly less cost-effective when adopting the societal perspective, which was driven by greater parent absenteeism cost and travel cost observed in the intervention arm. It is likely that parents/caregivers reported the time they took from work to attend the intervention sessions and the travel time to those sessions when completing the RUQ. Given that these costs were already included in the cost of intervention, the costs of the intervention from a societal perspective are likely overestimated due to potential double-counting.

There have only been two previous economic evaluation studies alongside a trial that examined early interventions for autistic children (Pye et al., 2023). One intervention (a social-communication program for parents in the UK) was deemed not cost-effective when provided in addition to usual care (Byford et al., 2015). A second intervention (training parents to implement applied behaviour analysis using telehealth) showed that telehealth delivery is an acceptable, cost-minimising alternative (Lindgren et al., 2016). However, direct comparisons to our study are challenging due to the use of different perspectives, study designs and outcome measures.

We used QALYs as a measure of outcome in our study, as recommended when conducting economic evaluations in autism (Tsiplova & Ungar, 2023). Although we reported parent/caregiver QALYs separately and combined those with child QALYs in the sensitivity analysis, our main analysis was based on child QALYs only, given that different instruments were used to generate QALYs (i.e., AQoL-4D and CHU9D). While it is common to use additive summation of spillover QALYs and patient QALYs, whether different weights should be applied to spillover QALYs compared with child QALYs is an area for further research (Al-Janabi et al., 2011). We did not find a significant difference in parent/caregiver QALYs between the intervention and TAU group, but greater QALY gains were observed for parents/caregivers than children in both groups. This confirms a previous study, which found that spillover QALYs gained by caregiver were of similar magnitude as the gains to the child when evaluating a sleep treatment for children with autism (Tilford et al., 2015). Although it is unclear whether gains in spillover QALYs can be attributed to the *Sleeping Sound* intervention, given the non-significance between the groups, it is important to consider and include spillover QALYs in future economic evaluation.

### Strengths and Limitations

This study has some key strengths and limitations. While previous economic evaluation studies of treatment interventions for autistic children have mainly used modelling techniques (Pye et al., 2023; Sampaio et al., 2021), we were able to collect cost-effectiveness data alongside effectiveness data. However, the follow-up period of 6 month employed in the trial may be considered as short, where long-term costs of outcomes remain unknown. A further limitation includes the proxy report of the CHU9D data in children above the age of 7 years, with evidence highlighting discrepancies in inter-rater agreement between child and proxy reporting (Khanna et al., 2022). There is also the need for a large-scale translation and implementation study of the *Sleeping Sound* intervention to inform its future roll-out and to determine implementation costs. In terms of generalizability, while trials usually have strict eligibility criteria, it is worth noting that children included in our study were recruited from a community sample, which increases generalisability. However, the exclusion of children with an intellectual disability can be considered a limitation, given that children with intellectual disability make up a relatively large proportion of autistic children (Howlin, 2000). Further, almost 1 in 5 caregivers were single parents and parents reported a broad range of education, further increasing generalisability. Additionally, compared with previous trial-based economic evaluations, our study is the largest to date with 245 children included in the trial. Another strength is the use of administrative MBS and PBS data to test the robustness of self-reported service use via the RUQ. Although self-reported service use is considered less reliable compared with administrative data, our sensitivity analysis results showed that use of MBS/PBS data resulted in greater ICERs but the intervention remained cost-effective, confirming a recent study that validated the use of self-reported service use (Dolar et al., 2023). However, despite the availability of MBS/PBS data, we had no access to hospital administrative data.

In conclusion, the *Sleeping Sound* intervention offers a cost-effective approach in improving sleep in primary school-aged autistic children. A pilot study of a telehealth version of the sleeping sound intervention has also recently been conducted. To inform the roll-out of the intervention, further evaluation of the implementation cost and alternative modes of administration (e.g., telehealth delivery) are worth considering.

## Supplementary Information

Below is the link to the electronic supplementary material.

Supplementary file1 (DOCX 57 KB)

## Data Availability

Data is not publicly available, as participants did not consent to the public use of their data.

## Footnotes

1 To reflect recent shifts in terminology among autistic people and their families, this paper will use the term “autism” in reference to Autism Spectrum Disorder (Bury et al., 2023). Further, the term “child/children with autism” will be used to reflect person-first language and “autistic child/children” to represent identity-first language preferences.
