# Supplementary material for: Sleeping Sound Autism Spectrum Disorder (ASD): Cost-Effectiveness of a Brief Behavioural Sleep Intervention in Primary School-Aged Autistic Children
Source: J Autism Dev Disord. 2024 Jun 4;55(9):3028–39. doi: 10.1007/s10803-024-06422-2 (PMC12367914; doi:10.1007/s10803-024-06422-2)
Supplement: Supplementary file 1 — Supplementary file1 (DOCX 57 KB) [file 10803_2024_6422_MOESM1_ESM.docx]

**Sleeping Sound Autism Spectrum Disorder (ASD): cost-effectiveness of a brief behavioural sleep intervention in primary school-aged autistic children**

**Supplementary Table 1: CHEERS checklist**

**Supplementary Table 2: Cost components by perspectives**

**Supplementary Table 3: Unit cost calculations, in Australian currency, 2016-2017 reference year**

**Supplementary Table 4: Pharmaceuticals unit costs, Australian currency, 2016-2017 reference year**

**Supplementary Table 5: Intervention cost**

**Supplementary Table 6: Missing values by treatment allocation**

**Supplementary Table 1: CHEERS checklist**

| **Topic** | **No.** | **Item** | **Location where item is reported** |
| --- | --- | --- | --- |
| **Title** |  |  |  |
|  | 1 | Identify the study as an economic evaluation and specify the interventions being compared. | Title |
| **Abstract** |  |  |  |
|  | 2 | Provide a structured summary that highlights context, key methods, results, and alternative analyses. | Abstract |
| **Introduction** |  |  |  |
| **Background and objectives** | 3 | Give the context for the study, the study question, and its practical relevance for decision making in policy or practice. | Introduction |
| **Methods** |  |  |  |
| **Health economic analysis plan** | 4 | Indicate whether a health economic analysis plan was developed and where available. | Methods |
| **Study population** | 5 | Describe characteristics of the study population (such as age range, demographics, socioeconomic, or clinical characteristics). | Trial design and participants |
| **Setting and location** | 6 | Provide relevant contextual information that may influence findings. | Trial design and participants |
| **Comparators** | 7 | Describe the interventions or strategies being compared and why chosen. | Interventions |
| **Perspective** | 8 | State the perspective(s) adopted by the study and why chosen. | Resource use and costs |
| **Time horizon** | 9 | State the time horizon for the study and why appropriate. | Introduction |
| **Discount rate** | 10 | Report the discount rate(s) and reason chosen. | Resource use and costs |
| **Selection of outcomes** | 11 | Describe what outcomes were used as the measure(s) of benefit(s) and harm(s). | Outcomes |
| **Measurement of outcomes** | 12 | Describe how outcomes used to capture benefit(s) and harm(s) were measured. | Outcomes |
| **Valuation of outcomes** | 13 | Describe the population and methods used to measure and value outcomes. | Outcomes |
| **Measurement and valuation of resources and costs** | 14 | Describe how costs were valued. | Resource use and costs |
| **Currency, price date, and conversion** | 15 | Report the dates of the estimated resource quantities and unit costs, plus the currency and year of conversion. | Resource use and costs |
| **Rationale and description of model** | 16 | If modelling is used, describe in detail and why used. Report if the model is publicly available and where it can be accessed. | N/A |
| **Analytics and assumptions** | 17 | Describe any methods for analysing or statistically transforming data, any extrapolation methods, and approaches for validating any model used. | Statistical analysis |
| **Characterising heterogeneity** | 18 | Describe any methods used for estimating how the results of the study vary for subgroups. | N/A |
| **Characterising distributional effects** | 19 | Describe how impacts are distributed across different individuals or adjustments made to reflect priority populations. | N/A |
| **Characterising uncertainty** | 20 | Describe methods to characterise any sources of uncertainty in the analysis. | Statistical analysis |
| **Approach to engagement with patients and others affected by the study** | 21 | Describe any approaches to engage patients or service recipients, the general public, communities, or stakeholders (such as clinicians or payers) in the design of the study. | N/A |
| **Results** |  |  |  |
| **Study parameters** | 22 | Report all analytic inputs (such as values, ranges, references) including uncertainty or distributional assumptions. | Results; Suppl Table 2 & 3 |
| **Summary of main results** | 23 | Report the mean values for the main categories of costs and outcomes of interest and summarise them in the most appropriate overall measure. | Results; Table 2 & 3 |
| **Effect of uncertainty** | 24 | Describe how uncertainty about analytic judgments, inputs, or projections affect findings. Report the effect of choice of discount rate and time horizon, if applicable. | Results; Table 3; Figure 1-4 |
| **Effect of engagement with patients and others affected by the study** | 25 | Report on any difference patient/service recipient, general public, community, or stakeholder involvement made to the approach or findings of the study | N/A |
| **Discussion** |  |  |  |
| **Study findings, limitations, generalisability, and current knowledge** | 26 | Report key findings, limitations, ethical or equity considerations not captured, and how these could affect patients, policy, or practice. | Discussion |
| **Other relevant information** |  |  |  |
| **Source of funding** | 27 | Describe how the study was funded and any role of the funder in the identification, design, conduct, and reporting of the analysis | Funding section |
| **Conflicts of interest** | 28 | Report authors conflicts of interest according to journal or International Committee of Medical Journal Editors requirements. | Conflicts of interest section |

*From:* Husereau D, Drummond M, Augustovski F, et al. Consolidated Health Economic Evaluation Reporting Standards 2022 (CHEERS 2022) Explanation and Elaboration: A Report of the ISPOR CHEERS II Good Practices Task Force. Value Health 2022;25. <doi:10.1016/j.jval.2021.10.008>

**Supplementary Table 2: Cost components by perspectives**

| **Cost category** | **Healthcare sector perspective** | **Societal perspective** |
| --- | --- | --- |
| Health professionals’ services | √ | √ |
| Pharmaceuticals | √ | √ |
| Emergency department visits | √ | √ |
| Hospitalisation | √ | √ |
| Ambulance | √ | √ |
| Out-of-pocket for health services | √ | √ |
| Transportation/travel |  | √ |
| Absenteeism |  | √ |

**Supplementary Table 3: Unit cost calculations, in Australian currency, 2016-2017 reference year**

| **Item name** | **Duration of consultation** | **Source** | **Unit** | **Unit cost** |
| --- | --- | --- | --- | --- |
| **Health professionals** | | | | |
| **General Practitioner** | <20 min, consulting room | Weighted average cost of MBS items 3, 23, 2712 | visit | $36.80 |
|  | 20-40 min, consulting room / other | Weighted average cost of MBS items 36, 2700, 2713, 2715, 2721, 2723 | visit | $72.76 |
|  | > 40 min consulting room, other | Weighted average cost of MBS items 44, 2701, 2717, 2725, 2727 | visit | $111.53 |
| **Psychologist** | <50 min | Weighted average cost of MBS items 10968, 80005, 80105 | visit | $76.78 |
|  | >30 min | Weighted average cost of MBS items 80000, 80005, 80105 | visit | $86.60 |
|  | >20 min | Weighted average cost of MBS items 80100, 80005, 80105 | visit | $64.81 |
|  | >50 min | Weighted average cost of MBS items 80010, 80015, 80110, 80115 | visit | $106.39 |
| **Paediatrician** | >10 min | Weighted average cost of MBS items 99,112 + 104,105,107,108,110,116,119,122,128,131 | visit | $65.94 |
|  | <10 min | Weighted average cost of MBS items 113, 114 + 104,105,107,108,110,116,119,122,128,131 | visit | $65.76 |
|  | >20 min | Weighted average cost of MBS items 133 + 104,105,107,108,110,116,119,122,128,131 | visit | $67.09 |
|  | >45 min | Weighted average cost of MBS items 132, 135, 137 + 104,105,107,108,110,116,119,122,128,131 | visit | $72.16 |
| **Speech Pathologist** | >20 min | Weighted average cost of MBS items 10970,81360 | visit | $57.54 |
|  | >50 min | Weighted average cost of MBS items 82005 | visit | $107.87 |
|  | >30 min | Weighted average cost of MBS items 82020 | visit | $87.02 |
| **Mental Health Nurse** | >20 min | Weighted average cost of MBS items 10956,82210 | visit | $34.92 |
|  | <20 min | Weighted average cost of MBS items 82205 | visit | $17.87 |
|  | >40 min | Weighted average cost of MBS items 82215 | visit | $50.45 |
| **Psychiatrist** | <15 min, consulting room/ other | Weighted average cost of MBS items 300, 310, 330 | visit | $41.29 |
|  | 15-30 min, consulting room/ other | Weighted average cost of MBS items 302, 312, 332 | visit | $28.15 |
|  | 30-45 min, consulting room/ other | Weighted average cost of MBS items 293, 304, 314, 334 | visit | $126.41 |
|  | 45-75 min, consulting room/ home/ other | Weighted average cost of MBS items 291, 296, 299, 306, 316, 319, 336 | visit | $207.90 |
|  | >75 minutes, consulting room/ other/ home | Weighted average cost of MBS items 308, 318, 338 | visit | $203.62 |
| **Social Worker** | >20 min | Weighted average cost of MBS items 80150,80155 | visit | $62.72 |
|  | >50 min | Weighted average cost of MBS items 80160,80165 | visit | $80.58 |
| **Occupational Therapist** | >20 min | Weighted average cost of MBS items 80125,80130,10958 | visit | $60.51 |
|  | >50 min | Weighted average cost of MBS items 80135,80140 | visit | $86.74 |
| **School counsellor** |  | Australian Bureau of Statistics, Data cube 11. All employees, Average weekly total cash earnings, average age - method of setting pay; by detailed occupation, 2018-19. https://www.abs.gov.au/statistics/labour/earnings-and-work-hours/employee-earnings-and-hours-australia/latest-release#data-download | per hour | $28.35 |
| **Hospital services** | | | | |
| **Hospital** | The same day/overnight | Sheet 10 Acute SD Overnight, NHCDC Cost report, round 21 | visit | $1408/$2338 |
| **Emergency department** | n/a | NHCDC Public Hospitals Cost Report, round 19 (2016/2017), sheet 15: https://www.ihpa.gov.au/what-we-do/nhcdc | visit | $450 |
| **Transportation** | | | | |
| **Car** * | n/a | The average vehicle in Victoria travelled 13,818 kilometres a year or 37.9 kilometres a day. (Budget direct). Average cents/km for medium vehicle is 70.8 (2019). Trip cost = 37.9 *0.708 = $26.83 (RACV website). CPI was applied to calculate cost for 2017 = 110.5/114.1 * 26.83 = $25.98 (ATO website) | trip | $25.98 |
| **Public transport (Concession)** | | Public transport Victoria Concession | trip | $4.20 |
| **Public transport (No concession)** | | Public transport Victoria myki daily full fare | trip | $8.20 |
| **Taxi** | n/a | Taxi Service commission. Metro taxi fares. Day rate (9am -5pm) = $4.20. Distance = $1.622/km. Assumption of average distance is 20km. $4.20+1.6222*20=36.644 (2020); https://cpv.vic.gov.au/passengers/taxi-fares/unbooked-fares-melbourne; derived on 14/01/2020. *CPI inflation formula* 110.5/116.6*36.644 =34.73 | trip | $34.73 |
| **Ambulance** | n/a | https://www2.health.vic.gov.au/hospitals-and-health-services/patient-care/ambulance-and-nept/ambulance-fees/. | trip | $1,206 |

* https://www.budgetdirect.com.au/car-insurance/research/average-kilometers-driven.html#:~:text=The%20average%20vehicle%20in%20Australia,492%2C495%20kilometres%20travelled%20each%20day. https://www.racv.com.au/on-the-road/buying-a-car/car-running-costs.html. https://www.ato.gov.au/Rates/Consumer-price-index/; n/a not applicable.

**Supplementary Table 4: Pharmaceuticals unit costs, Australian currency, 2016-2017 reference year**

| **Pharmaceuticals** | **Dosage** | **Pack qty** | **DPMQ** | **General Patient Charge** | **Reimbursement** | **Reimbursement with concession** |
| --- | --- | --- | --- | --- | --- | --- |
| **Ritalin 10, mg** | 10 | 100 | $21.04 | $26.61 | -$5.57 | $14.74 |
| **Ritalin LA, mg** | 10 | 30 | $35.18 | $38.80 | -$3.62 | $28.88 |
|  | 20 | 30 | $44.45 | $38.80 | $5.65 | $38.15 |
|  | 30 | 30 | $51.91 | $38.80 | $13.11 | $45.61 |
|  | 40 | 30 | $54.44 | $38.80 | $15.64 | $48.14 |
| **Concerta, mg** | 18 | 30 | $51.20 | $38.80 | $12.40 | $44.90 |
|  | 27 | 30 | $55.34 | $38.80 | $16.54 | $49.04 |
|  | 36 | 30 | $59.47 | $38.80 | $20.67 | $53.17 |
|  | 54 | 30 | $68.62 | $38.80 | $29.82 | $62.32 |
| **Dexamphetamine, mg** | 5 | 100 | $20.99 | $26.56 | -$5.57 | $14.69 |
| **Strattera, mg** | 10 | 28 | $167.58 | $38.80 | $128.78 | $161.28 |
|  | 100 | 28 | $112.62 | $38.80 | $73.82 | $106.32 |
|  | 18 | 28 | $167.58 | $38.80 | $128.78 | $161.28 |
|  | 25 | 28 | $167.58 | $38.80 | $128.78 | $161.28 |
|  | 40 | 28 | $167.58 | $38.80 | $128.78 | $161.28 |
|  | 60 | 28 | $167.58 | $38.80 | $128.78 | $161.28 |
|  | 80 | 28 | $112.62 | $38.80 | $73.82 | $106.32 |
| **Catapres, microgram** | 100 | 100 | $26.14 | $31.71 | -$5.57 | $19.84 |
|  | 150 | 100 | $32.09 | $37.66 | -$5.57 | $25.79 |
| **Risperdal** | 2 mg | 60 | $26.52 | $32.09 | -$5.57 | $20.22 |
|  | 500 microg | 60 | $17.69 | $23.27 | -$5.58 | $11.39 |
|  | 1 mg/ml oral liquid | 100 ml | $112.02 | $38.80 | $73.22 | $105.72 |
|  | 37.5 mg injection | 1 | $344.80 | $38.80 | $306.00 | $338.50 |
|  | 3 mg | 60 | $35.16 | $38.80 | -$3.64 | $28.86 |
|  | 25 mg injection | 1 | $265.78 | $38.80 | $226.98 | $259.48 |
|  | 1 mg | 60 | $17.54 | $23.11 | -$5.57 | $11.24 |
|  | 50 mg injection | 1 | $422.98 | $38.80 | $384.18 | $416.68 |
|  | 500 microg | 20 | $17.70 | $23.27 | -$5.57 | $11.40 |
|  | 4 mg | 60 | $43.77 | $38.80 | $4.97 | $37.47 |
| **Prozac, mg** | 20 | 28 | $12.35 | $21.02 | -$8.67 | $6.05 |
| **Lovan, mg** | 20 | 28 | $14.27 | $19.84 | -$5.57 | $7.97 |
| **Circadin*** | 2 mg | 240 | n/a | $35.74 | n/a | n/a |
|  | 2.5mg | 240 | n/a | $36.91 | n/a | n/a |
|  | 5 mg | 60 | n/a | $13.38 | n/a | n/a |
|  | 3 mg | 60 | n/a | $6.34 | n/a | n/a |
|  | 10 mg | 60 | n/a | $15.05 | n/a | n/a |
|  | 20 mg | 60 | n/a | $66.39 | n/a | n/a |
|  | 1 mg | 60 | n/a | $6.28 | n/a | n/a |
|  | 1.5 | 60 | n/a | $28.40 | n/a | n/a |
| **Circadin liquid**** | 3 mg/1ml | 55 | n/a | $16.43 | n/a | n/a |
| **Vyvanse, mg** | 50 | 30 | $128.11 | $45.00 | $83.11 | $121.81 |
|  | 30 | 30 | $128.11 | $45.00 | $83.11 | $121.81 |
|  | 70 | 30 | $128.11 | $45.00 | $83.11 | $121.81 |
| **Fish oil, mg** | 1000 | 200 | n/a | $10.97 | n/a | n/a |
| **Fish oil, ml** *** | 5 | 500 | n/a | $41.70 | n/a | n/a |

* https://au.iherb.com/search?kw=melatonin&p=1, derived Aug 2020; ** https://au.iherb.com/pr/KAL-Melatonin-Natural-Raspberry-Flavor-3-mg-1-85-fl-oz-55-ml/86393?gclid=EAIaIQobChMI9cuPhqGm6wIVcdOWCh3WgQQLEAQYAiABEgLKhfD_BwE&gclsrc=aw.ds derived on 19/08/20; *** https://www.chemistwarehouse.com.au/buy/56504/ethical-nutrients-high-strength-omega-3-liquid-(fruit-punch)-170ml; n/a not applicable; DPMQ Dispensed Price for Maximum Quantity; qty quantity.

**Supplementary Table 5: Intervention cost**

| ***Resource*** | ***Unit*** | ***Amount*** | ***Unit cost*** | ***Total cost (n=123)*** | ***Comments*** | ***Healthcare perspective*** | ***Societal perspective*** |
| --- | --- | --- | --- | --- | --- | --- | --- |
| **Development of the SSA intervention content (included in sensitivity analysis only)** | | | | | | | |
| Content modification | per hour | 30 | $200.00 | $6,000.00 | APS rate for clinical psychologist | 🗸 | 🗸 |
| Graphic designer/ social stories | n/a | n/a | n/a | $5,000.00 | One-off payment |  |  |
| Updating documents | per hour | 8 | $42.46 | $339.68 | Casual RA rate |  |  |
| Total development cost |  |  |  | **$11,339.68** |  |  |  |
| **Training (2 sessions for 3 hours)** | | | | | | | |
| Trainer time - clinical psychologist | per hour | 6 | $200.00 | $1,200.00 | APS rate for clinical psychologist 2 x 3h sessions | 🗸 | 🗸 |
| Trainee time - general psychologist | per hour | 18 | $185.00 | $3,330.00 | APS rate for general psychologist 2 x 3h sessions x 3 trainees |  |  |
| Training preparation - clinical psychologist | per hour | 16 | $200.00 | $3,200.00 | APS rate for clinical psychologist |  |  |
| Trainee familiarising self with material | per hour | 10.5 | $185.00 | $1,942.50 | APS rate for general psychologist 3.5h per trainee |  |  |
| Total training time |  |  |  | **$9,672.50** |  |  |  |
| **Materials** | | | | | | | |
| Printing cost (clinician manual, participant material) | n/a | n/a | n/a | $219.29 | Total printing cost | 🗸 | 🗸 |
| Clinician manuals/folders - assembling | per hour | 4 | $42.46 | $169.84 | Casual RA rate |  |  |
| Clinician manuals/folders - supervision | per hour | 2 | $185.00 | $370.00 | APS rate for general psychologist |  |  |
| Participants manuals/folder - assembling | per hour | 8 | $42.46 | $339.68 | Casual RA rate |  |  |
| Total |  |  |  | **$1,098.81** |  |  |  |
| **Intervention delivery - intended** | | | | | | | |
| 2 face-to-face sessions 50min + 10 min prep time and case notes | per hour | 246 | $185.00 | $45,510.00 | 123 x 2sessions = 246 hours | 🗸 | 🗸 |
| Travel time - clinician | per hour | 246 | $138.75 | $34,132.50 | 45 min travel ($185 per hour): $138.75 |  |  |
| Travel cost - clinician (cooperate car) | per trip | 246 | $19.80 | $4,870.80 | Average travel distance: 30km ATO cents per km: $0.66 (2016/2017) 30km x $0.66 = $19.8 per trip |  |  |
| Phone calls (2 weeks after) 50min + 10 min prep time and case notes | per call | 123 | $185.00 | $22,755.00 | APS rate for general psychologist |  |  |
| Scheduling time | per call | 123 | $21.23 | $2,611.29 | 30min by RA ($42.46 p/h) $21.23 |  |  |
| Reminder texts | per text | 369 | $0.10 | $36.90 | 123 x 3 session = 369 |  |  |
| Total |  |  |  | **$109,916.49** |  |  |  |
| **Intervention delivery - actual** | | | | | | | |
| Face-to-face session - actual time | per hour | 192 | $185.00 | $35,520.00 | Average session 1: 51.81 min; Average session 2: 32.54 min Total: 192 hours and 10 mins | 🗸 | 🗸 |
| Phone calls - actual time | per hour | 42 | $185.00 | $7,770.00 | Average session 3: 16.86 min; Total: 41 hours 45 mins |  |  |
| Travel time - clinician | per hour | 246 | $138.75 | $34,132.50 | 45 min travel ($185 per hour): $138.75 |  |  |
| Travel cost - clinician (cooperate car) | per trip | 246 | $19.80 | $4,870.80 | Average travel distance: 30km ATO cents per km: $0.66 (2016/2017) 30km x $0.66 = $19.8 per trip |  |  |
| Scheduling time | per call | 123 | $21.23 | $2,611.29 | 30min by RA ($42.46 p/h) $21.23 |  |  |
| Reminder texts | per text | 369 | $0.10 | $36.90 | 123 x 3 session = 369 |  |  |
| Total |  |  |  | **$84,941.49** |  |  |  |
| **Supervision (1 x 1h fortnightly session)** | | | | | | | |
| Supervisor | per hour | 49 | $200.00 | $9,800.00 | APS rate for clinical psychologist | 🗸 | 🗸 |
| Clinician | per hour | 147 | $185.00 | $27,195.00 | 49h x 3 clinician = 147h |  |  |
| Total |  |  |  | **$36,995.00** |  |  |  |
| **Cost for participants** | | | | | | | |
| Parent time to attend all sessions | per hour | 615 | $31.36 | $19,286.40 | 50min per session x 3 = 150min (2.5h) 2.5h x 123 x 2 session= 615h National average weekly earnings: 1191.50/38h=$31.36 |  | 🗸 |
| Travel time - parent | per participant | 123 | $31.36 | $3,857.28 | average time for 2 sessions ~ 69.18 min |  |  |
| Travel cost - parent | per participant | 123 | $47.00 | $5,781.00 | Average km for 2 sessions ~ 71.24km ATO cents per km: $0.66 (2016/2017) 71.24km x $0.66 = $47 per trip |  |  |
| Parking for participants | per permit | 246 | $8.00 | $1,968.00 |  |  |  |
| Time for homework | per participant | 123 | $2.61 | $321.03 | 5mins of $31.36p/h = $2.61 |  |  |
| Total |  |  |  | **$31,213.71** |  |  |  |
| **TOTAL cost – actual delivery time** | | | | | | | |
| Total for 123 participants |  |  |  |  |  | $132,707.80 | $163,921.51 |
| Cost per participant |  |  |  |  |  | **$1,079** | **$1,333** |
| **TOTAL cost – intended delivery time** | | | | | | | |
| Total for 123 participants |  |  |  |  |  | $157,682.80 | $188,896.51 |
| Cost per participant |  |  |  |  |  | **$1,282** | **$1,536** |

**Supplementary Table 6: Missing values by treatment allocation**

|  | **Intervention (n=123)** | | **TAU (n=122)** | |
| --- | --- | --- | --- | --- |
|  | **N Missing** | **% Missing** | **N Missing** | **% Missing** |
| **3-months follow up** | | | | |
| **Health professionals** | 21 | 17.07 | 25 | 20.49 |
| **Pharmaceuticals** | 21 | 17.07 | 25 | 20.49 |
| **Hospital services** | 21 | 17.07 | 25 | 20.49 |
| **Out-of-pocket** | 21 | 17.07 | 25 | 20.49 |
| **Time cost** | 21 | 17.07 | 25 | 20.49 |
| **Travel cost** | 21 | 17.07 | 25 | 20.49 |
| **CHU9D 3m** | 21 | 17.07 | 25 | 20.49 |
| **AQoL-4D 3m** | 21 | 17.07 | 25 | 20.49 |
| **6-months follow up** | | | | |
| **Health professionals** | 33 | 26.83 | 34 | 27.87 |
| **Pharmaceuticals** | 33 | 26.83 | 34 | 27.87 |
| **Hospital services** | 33 | 26.83 | 34 | 27.87 |
| **Out-of-pocket** | 33 | 26.83 | 34 | 27.87 |
| **Time cost** | 33 | 26.83 | 34 | 27.87 |
| **Travel cost** | 33 | 26.83 | 34 | 27.87 |
| **CHU9D 6m** | 33 | 26.83 | 34 | 27.87 |
| **AQoL-4D 6m** | 33 | 26.83 | 34 | 27.87 |
| **MBS and PBS data** | | | | |
| **MBS data** | 35 | 28.46 | 44 | 36.07 |
| **PBS data** | 44 | 35.78 | 50 | 40.98 |
